# Characterization of an enterococcal phage endolysin as a potential antimicrobial agent against *Streptococcus suis*

**DOI:** 10.1186/s13567-025-01608-7

**Published:** 2025-09-02

**Authors:** Jing Wang, Yaowei Liu, Siyu Liang, Xiaofeng Lu, Qiu Xu, Cuilong Fan, Wanjiang Zhang, Siguo Liu, Fang Xie

**Affiliations:** State Key Laboratory for Animal Disease Control and Prevention, Harbin Veterinary Research Institute, Chinese Academy of Agricultural Sciences, Harbin, China

**Keywords:** *Streptococcus suis*, phage, endolysin, antibacterial activity, anti-biofilm activity

## Abstract

*Streptococcus suis*, an important zoonotic pathogen capable of transmission from pigs to humans, represents a critical threat to both public health and the global pork industry. The increasing prevalence of multidrug-resistant *S. suis* strains, coupled with their ability to form biofilms, has necessitated the development of alternative antimicrobial strategies. In this study, we characterized the therapeutic potential of Ply113, an endolysin derived from an *Enterococcus faecium* phage, against *S. suis*. Ply113 has shown potent bactericidal activity against *S. suis* in vitro, with rapid time-kill characteristics and broad-spectrum efficiency against clinically prevalent serotypes (2, 3, 4, 7, and 9). Transmission electron microscopy analysis revealed that Ply113 induced distinct morphological alterations in *S. suis*, including cell wall disintegration and cytoplasmic leakage. This endolysin exhibited anti-biofilm functionality, eradicating biofilms formed by clinical strain of *S. suis* in a concentration-dependent manner. In murine models of bacteremia, a single administration of Ply113 provided complete protection against lethal *S. suis* infection, significantly decreasing the bacterial burden in the liver and spleen and attenuating organ injury. Additionally, Ply113 has been shown to be safe for mice, with no adverse effects. Taken together, our findings indicate that Ply113 is a promising alternative antimicrobial agent for combating biofilm-related infections caused by *S. suis*.

## Introduction

*Streptococcus suis* is a Gram-positive bacterium that causes clinical conditions such as septicemia, meningitis, arthritis, endocarditis, and pneumonia in pigs, which is responsible for considerable economic loss in the porcine industry [1]. *S. suis* is the most frequently isolated bacterial pathogen in pig farms globally, and its prevalence exceeds 80% in some countries and regions [2–5]. As an emerging zoonotic pathogen, *S. suis* can be transmitted to humans via the consumption of contaminated pork products or close contact with sick or carrier pigs. *S. suis* poses a significantly high risk to humans, especially individuals with compromised immune systems*,* leading to meningitis and streptococcal toxic shock-like syndrome, which are frequently accompanied by high mortality rates in severe cases or lifelong sequelae following survival, such as deafness [6, 7]. Although human *S. suis* infections are considered sporadic, two large-scale outbreaks of human infections have been documented in China, indicating the public health threat posed by this pathogen [8, 9].

According to the antigenic differences in the capsular polysaccharide (CPS), *S. suis* can be categorized into 29 serotypes (1–19, 21, 23–25, 27–31, 1/2) [10]. Although the distribution of *S. suis* serotypes from clinical cases may differ across geographic locations, *S. suis* serotype 2 is commonly regarded as the most prevalent serotype worldwide and is associated with the majority of severe infections in pigs and humans, particularly in Asian countries [11]. Additionally, serotypes 3, 4, 7, and 9 are the predominant prevalent serotypes worldwide [1, 10]. Currently, antibiotics remain the mainstay treatment options for managing *S. suis* infections. However, the emergence of antibiotic-resistant strains of *S. suis*, especially multidrug-resistant isolates, has made controlling these infections a major challenge in the pig industry [12]. Although vaccines may represent a rational alternative to antibiotics, the high genetic and phenotypic variability of *S. suis* poses significant difficulties in the development of a universal vaccine [13, 14]. Given these challenges, there is an urgent need to develop novel antimicrobial approaches against the growing threat posed by antibiotic-resistant strains of *S. suis*.

Endolysins are phage-derived enzymatic proteins biosynthesized during the terminal phase of the phage replication cycle that function to degrade bacterial peptidoglycan structures and facilitate the release of progeny phages [15]. Thus, exogenous delivery of purified endolysins to peptidoglycan substrates induces “lysis from without” with high bactericidal efficiency and has sparked interest as a prospective antibacterial agent [16]. The evolutionary conservation of peptidoglycan as the enzymatic substrate for endolysins confers a remarkably low possibility of resistance development, which is a critical advantage in combating multidrug-resistant bacteria [17]. Various clinical trials have demonstrated the safety and efficacy of phage endolysins, which aligns with extensive evidence from animal models [18]. Furthermore, endolysins with enhanced stability and broad-spectrum bactericidal activity show increased potential for clinical application [18, 19]. To date, only temperate phages infecting *S. suis* have been isolated [20, 21], limiting the development of phages as a therapeutic approach against *S. suis*. Thus, it is critical to explore the possible application of phage-derived endolysins as the alternative antimicrobial agent for combating *S. suis*.

Our previous study identified the endolysin Ply113 from *Enterococcus faecium* phage and demonstrated its superior bactericidal activity against *E. faecium*, *Enterococcus faecalis, and Staphylococcus aureus*. Ply113 contains an N-terminal NlpC/P60 catalytic domain and a C-terminal SH3b domain for cell wall binding. Moreover, Ply113 exhibited favorable stability under various temperature and salinity conditions, indicating its potential as a promising antimicrobial agent [22]. In the present study, we investigated the bactericidal activity of Ply113 against *S. suis* in vitro. Moreover, the effects of Ply113 on *S. suis* biofilm disruption were assessed. The therapeutic efficacy of Ply113 against lethal *S. suis* infection in vivo was further evaluated in a murine bacteremia model.

## Materials and methods

### Bacterial strains and protein expression

All *S. suis* strains used in this study were routinely grown at 37 °C under 5% CO_2_ in tryptone soy broth (TSB) or tryptone soy agar (TSA; Bacto™, Becton Dickinson) supplemented with 5% newborn bovine serum unless otherwise specified. The *S. suis* 05ZYH33 strain, a highly virulent strain of serotype 2, was isolated from a clinical patient during the 2005 Sichuan outbreak in China (GenBank™ accession no. CP000407.1). Other *S. suis* isolates were obtained from different diseased pigs and were identified as serotype 2 (6B, 449 T, S8, S123, JL8F strains), serotype 3 (1518, A21, HLJ01, F32), serotype 4 (M17, NMG02, Y8, GN12), serotype 7 (NY7, G62, P76, L10), and serotype 9 (HN21, GZ-2, NF01, E05) using 16S rRNA sequencing analysis and serotyping as described previously [23]. The Ply113 endolysin was expressed in the *Escherichia coli* strain BL21(DE3) and purified via Ni-affinity chromatography as described previously [22].

### Bactericidal assay

The bactericidal activity of Ply113 against *S. suis* strains was measured as a reduction in colony-forming unit (CFU) as previously described [24]. *S. suis* was grown at 37 °C until it reached the logarithmic growth phase. The bacterial culture was centrifuged at 3500 × *g* for 10 min, washed three times, and then resuspended in sterile phosphate-buffered saline (PBS, pH 7.4). The concentration was adjusted to approximately 5 × 10^7^ CFU/mL. To assess the impact of concentration on the bactericidal activity of Ply113 endolysin, bacterial suspensions of *the S. suis* 05ZYH33 strain were treated with Ply113 at different concentrations (0–16 µg/mL) for 1 h at 37 °C. For time-kill assays, bacterial suspensions of the *S. suis* 05ZYH33 strain were treated with 16 µg/mL Ply113 for different durations (0–60 min) at 37 °C. The samples were serially diluted tenfold in PBS before being distributed onto TSA plates for determination of viable counts. As a negative control, bacteria were treated with PBS under the same conditions. The experiments were performed in technical triplicates.

### Determination of Ply113 antimicrobial spectrum

CFU reduction assays were used to determine the lytic activity of Ply113 against different *S. suis* strains. Briefly, the strains were cultured at 37 °C until they reached an optical density of 0.6 at 600 nm (OD_600_ nm), after which they were washed and resuspended in sterile PBS. Ply113 was added to the bacterial solution at a final concentration of 16 µg/mL and incubated at 37 °C for 1 h. As a negative control, bacteria were treated with PBS buffer under the same conditions. The experiment was performed in technical triplicates.

### Transmission electron microscopy

The *S. suis* 05ZYH33 strain was grown at 37 °C until the logarithmic growth phase was reached. *S. suis* cells were washed with PBS and treated with different concentrations of Ply113 (0–4 µg/mL) for 30 min at 37 °C. Bacterial cells treated with PBS under the same conditions were used as a negative control. The bacterial cells were fixed with glutaraldehyde for 12 h at 4 °C, incubated with 1% osmium tetroxide for 2 h at 4 °C, and dehydrated in acetone. The fixed samples were subsequently immersed in epoxy resin for an overnight period and polymerized at high temperature for 48 h. Ultrathin Sections (65–70 nm thick) were sliced and placed on copper grids. Following staining with uranyl acetate and lead citrate, changes in bacterial morphology were observed using a transmission electron microscopy (Hitachi, Tokyo, Japan).

### Biofilm assays

A crystal violet assay was used to assess the effectiveness of Ply113 on *S. suis* biofilm as previously described [25]. *S. suis* JL8F, a strong biofilm-forming strain, was grown in TSB medium supplemented with 5% newborn bovine serum until it reached the logarithmic growth phase and then diluted 100-fold in TSB medium. One hundred microliters of bacterial solution (10^6^ CFU/mL) was added to each well of a 96-well polypropylene microtiter plate (Corning, NY, USA) and incubated at 37 °C for 24 h to allow biofilm formation. The wells were washed with PBS to remove the planktonic cells. Next, the preformed biofilms were treated with different concentrations of Ply113 (0–16 μg/mL). Following incubation for 2 h, each well was carefully washed with PBS, and the residual biofilms were fixed with methanol and stained with 1% crystal violet for 15 min. After staining, the attached crystal violet was dissolved in 33% glacial acetic acid. Biomass was quantified by measuring absorbance of each well at OD_570 nm_ using an EnVision Multimode Plate Reader (PerkinElmer, Waltham, USA). To assess the bactericidal efficiency of Ply113 against established biofilms of *S. suis*, the quantification of viable cells in biofilm after 2 h of Ply113 treatment was performed using plate colony counts [26]. The experiment was performed in technical triplicates with three biological replicates for each sample.

### Confocal laser scanning microscopy

The biofilm samples for confocal laser scanning microscopy (CLSM) observation were prepared according to a previous procedure with some modifications [27]. A total of 2 × 10^6^ CFU of *S. suis* JL8F was added to glass-bottom microwell dishes (Nest, Wuxi, China). Following 24 h of incubation at 37 °C, the biofilms were washed to remove the planktonic cells and treated with Ply113 at a concentration of 16 μg/mL and further incubated for 2 h. Mature biofilms treated with PBS served as control. The biofilms were subsequently washed and stained for 20 min in the dark at room temperature with a LIVE/DEAD™ BacLight™ Bacterial Viability Kit (Invitrogen™, Thermo Fisher, USA). For exopolysaccharide visualization, biofilms were stained with Alexa Fluor 350-labeled Concanavalin A (Invitrogen™, Thermo Fisher, USA). Biofilm images were acquired using a confocal scanning laser microscope (Zeiss LSM 800, Jena, Germany). Quantification of biofilm was analyzed for the bio-volume and thickness using the COMSTAT software [28]. Quantification of area stained for exopolysaccharide was performed using ImageJ software [29].

### Experimental animals

All the mouse experiments were conducted according to the Guide for the Care and Use of Laboratory Animals of the Ministry of Science and Technology of the People’s Republic of China. The procedure for the animal experiments in this study was approved by the Animal Ethics Committee of the Harbin Veterinary Research Institute of the Chinese Academy of Agricultural Sciences (Approval No. HVRI-IACUC-23121901GR). Specific-pathogen-free (SPF)-grade C57BL/6 mice (6 weeks old) were procured from Vital River Experimental Animal Technology Co., Ltd. (Beijing, China) and housed under a 12-h light/dark cycle in individually ventilated cages with free access to standard animal food and water.

### Toxicity to C57BL/6 mice

Fifteen 6-week-old C57BL/6 mice were randomly divided into three groups, with five mice in each group (*n* = 5). In group 1, five mice received an intraperitoneal injection of 8 mg/kg Ply113 in 100 µL of PBS, whereas five mice in group 2 received an injection of 16 mg/kg Ply113. The control group was injected with 100 µL of PBS. After administration, the mice in each group were observed for clinical signs, such as lethargy, loss of appetite, ruffled fur, hunched posture, paralysis, and weighed daily for 10 days. After that, the mice were euthanized, and the organs were collected to examine changes in gross lesions.

### Therapeutic effect of Ply113 in vivo

A murine bacteremia model of *S. suis* was constructed as previously described with some modifications [30]. Briefly, C57BL/6 mice (6 weeks old) were injected intraperitoneally with 100 μL of 8 × 10^8^ CFU log-phase *S. suis* 05ZYH33, and then the mice were randomly divided into four groups, with ten mice in each group (*n* = 10). Two hours after infection, groups 1, 2, and 3 were administered Ply113 at therapeutic doses of 2, 4, and 8 mg/kg, respectively. The control group was injected with 100 µL of PBS. Mortality following intraperitoneal challenge was monitored for 7 days.

For bacterial load detection, C57BL/6 mice (6 weeks old) were infected with 8 × 10^7^ CFU of *S. suis* 05ZYH33 and then administered 8 mg/kg Ply113 at 2 h post-infection. The control group was administered 100 µL of PBS. The mice in each group were euthanized at 24 h after infection, and the liver and spleen were aseptically removed, weighed and homogenized. The homogenate was then serially diluted in PBS and plated on TSA plates to calculate the number of bacterial CFUs per milliliter in each sample. For histopathological analysis, the liver and spleen were removed and fixed in 4% paraformaldehyde. The dehydrated tissues were embedded in paraffin and subsequently further sliced into 4 μm-thick sections. The paraffin sections were dewaxed and stained with hematoxylin and eosin (H&E). The pathological changes in each tissue sample were evaluated using light microscopy.

### Statistical analysis

GraphPad Prism 9.0 (GraphPad Software, San Diego, CA) was used for statistical analysis and graphing. The data are expressed as the mean ± standard deviation (SD). Statistical significance was determined via one-way analysis of variance (ANOVA) or an unpaired *t* test. *P* < 0.05 was considered significant (*P < 0.05; **P < 0.01; ***P < 0.001).

## Results

### Ply113 displays bactericidal activity against *S. suis*

**Figure 1:**
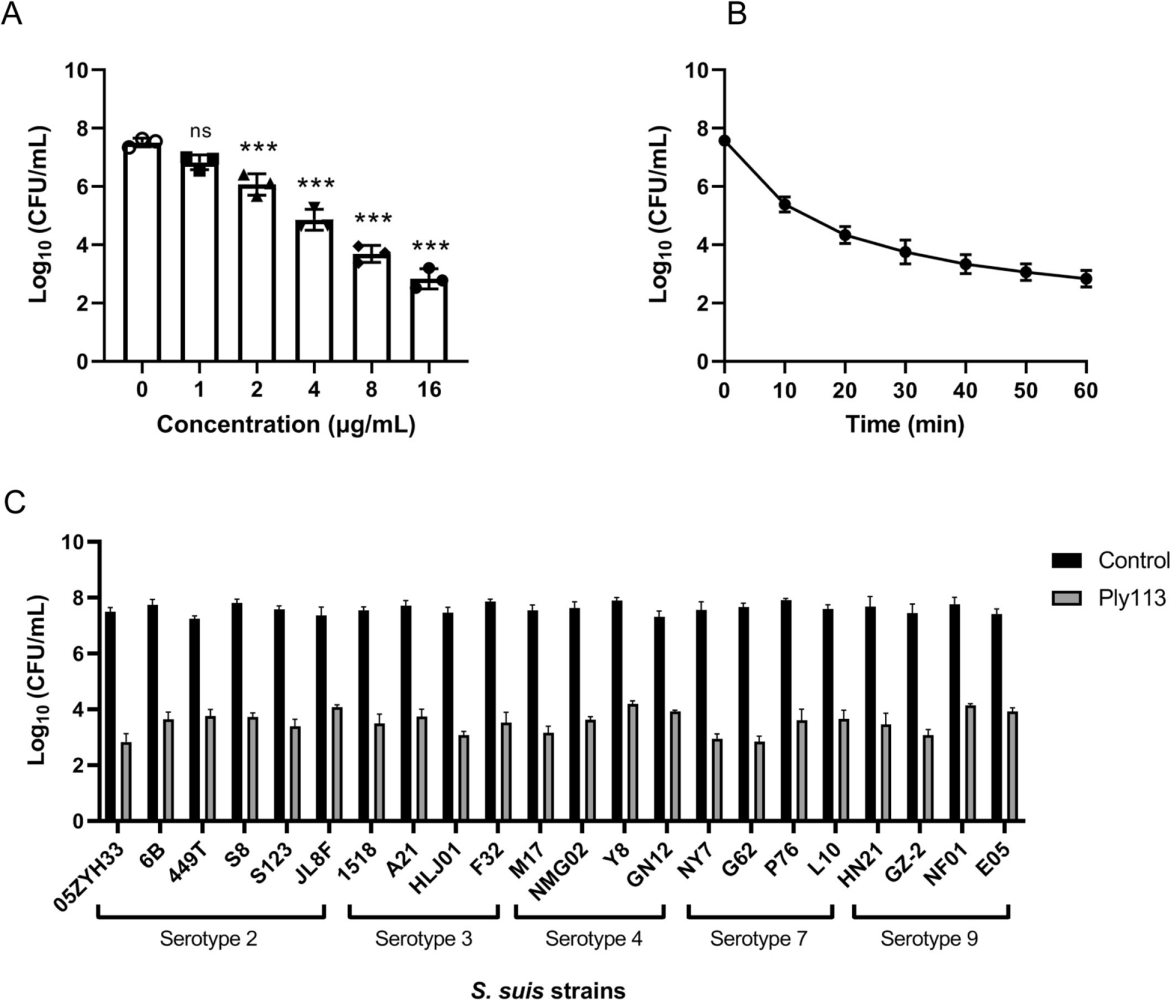
**Lytic spectrum and bactericidal activity of Ply113 against** ***S. suis***. **A** Concentration-dependent killing efficacy of Ply113. Bacterial cells were treated with various concentrations of Ply113 (0–16 μg/mL) for 60 min before being diluted and plated on TSA plates for CFU counting. The data are presented as the mean ± SD from three independent tests. Statistical significance was calculated via one-way analysis of variance (ANOVA). ***p < 0.001; ns, not significant. **B** Time-dependent killing curves of Ply113. The bacterial cells were treated with 16 μg/mL Ply113 for different durations before being diluted and plated on TSA plates for CFU counting. **C** Bactericidal activities of Ply113 against various serotypes of *S. suis*. Each strain was treated with 16 μg/mL Ply113 or PBS for 60 min. The reduction in bacterial CFUs was used to assess the bactericidal efficacy of Ply113 against *S. suis* strains.

To evaluate the bactericidal activity of Ply113 in vitro, various concentrations of Ply113 (0–16 µg/mL) were incubated with *S. suis*, and the number of viable bacteria was calculated. As shown in Figure 1A, Ply 113 caused a gradual decrease in the number of viable bacteria in a dose-dependent manner, with a 1.44 log_10_ reduction at 2 µg/mL and a 4.68 log_10_ reduction at 16 μg/mL. The results of the time-killing assay also revealed the effective bactericidal activity of Ply113 against *S. suis* within 60 min at a concentration of 16 μg/mL (Figure 1B). The number of viable bacteria decreased by 2.20 log_10_ within 10 min and 3.83 log_10_ within 30 min. After treatment for 60 min, Ply113 caused a reduction of 4.74 log_10_ (Figure 1B). The bactericidal activities of Ply113 against the main prevalent serotypes of *S. suis* isolates, including serotypes 2, 3, 4, 7, and 9, were subsequently determined in vitro. As shown in Figure 1C, despite variations in lytic efficiency, Ply113 was able to kill all *S. suis* strains used in this study (22/22), exhibiting broad bactericidal activity. These results suggest that Ply113 is highly active against *S. suis* strains in vitro.

### Ply113 destroys the cell morphology of *S. suis*

**Figure 2:**
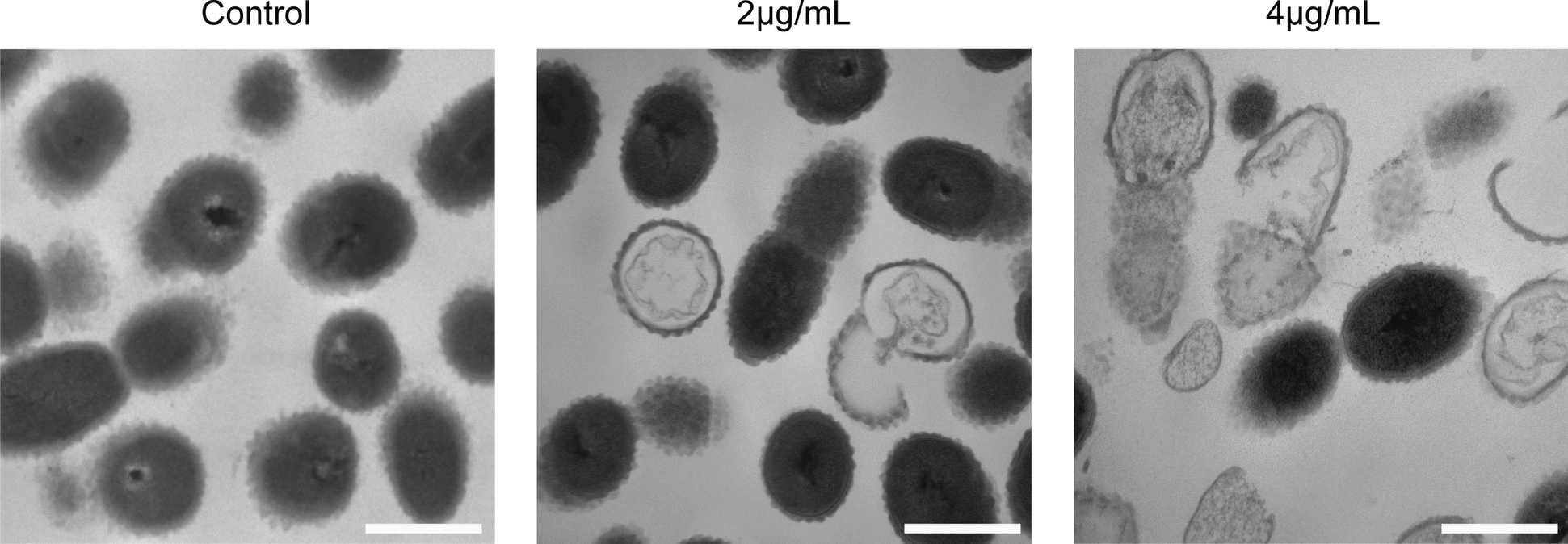
**Morphological changes in** ***S. suis* after Ply113 treatment**. TEM analysis of *S. suis* 05ZYH33 in the logarithmic phase or after exposure to various concentrations of Ply113 (2 and 4 μg/mL) for 30 min at 37 °C. Scale bar, 500 μm.

To investigate the effects of Ply113 on the cell morphology of *S. suis*, the bacterial suspensions of the *S. suis* 05ZYH33 strain were treated with Ply113 at lower concentrations (0–4 µg/mL) and observed via transmission electron microscopy. As shown in Figure 2, *S. suis* 05ZYH33 treated with PBS displayed an intact spherical shape with well-defined cell envelope structures. However, evident cell perforation and deformation were observed in *S. suis* treated with Ply113. At 2 µg/mL, Ply113 was found to induce rupture of the cytoplasmic membrane and cell wall, resulting in a modest number of bacterial ghosts devoid of cytoplasmic components (Figure 2). Increasing the concentration of Ply113 to 4 µg/mL led to a greater proportion of bacterial ghosts and more severe ultrastructural damage (Figure 2).

### Ply113 exhibits anti-biofilm activity against *S. suis*

**Figure 3:**
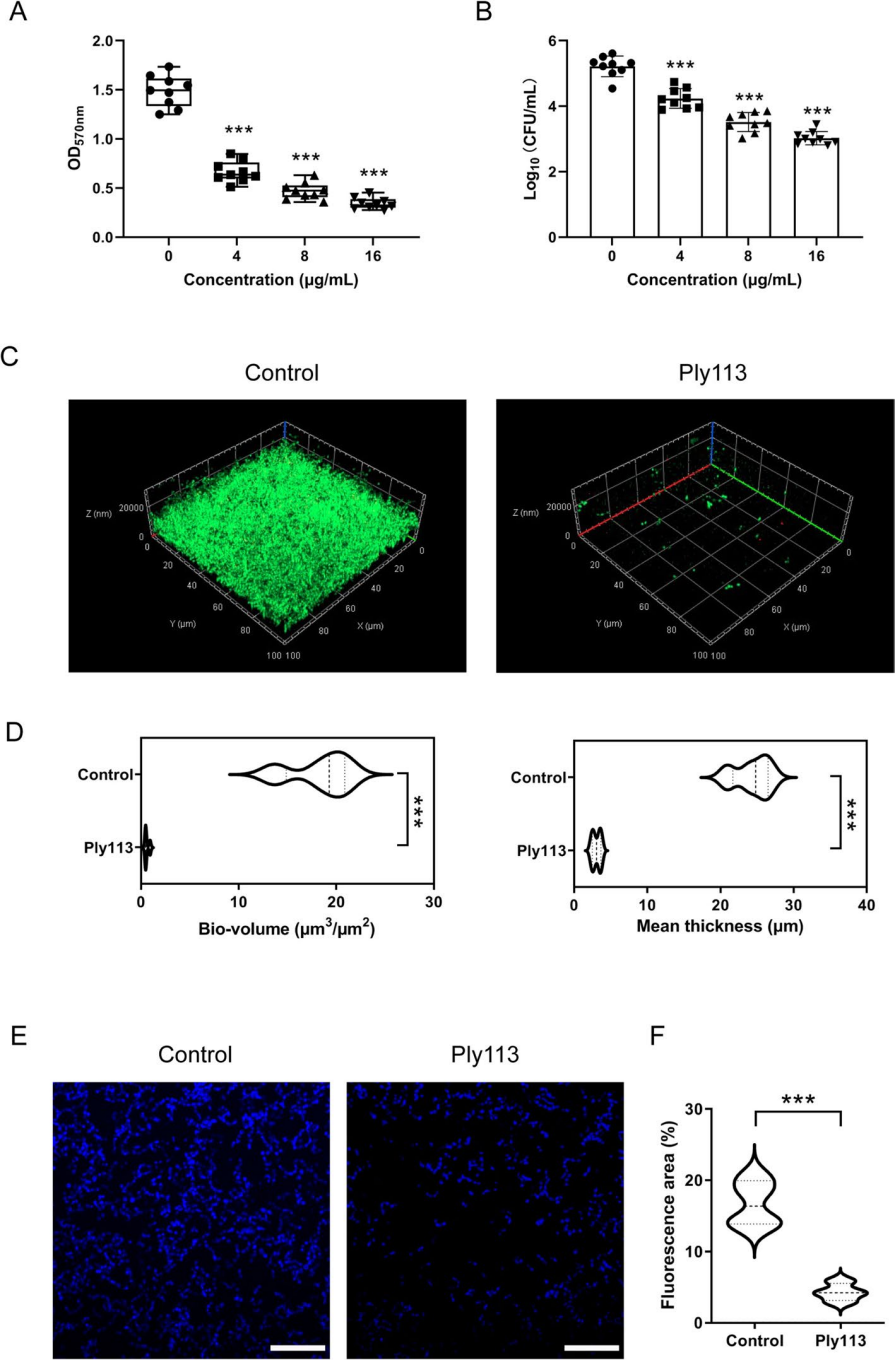
**Effects of Ply113 on** ***S. suis* established biofilms**. **A** Ply113 antibiofilm activity quantification by crystal violet staining. The preformed *S. suis* biofilms were treated with different concentrations of Ply113 (0–16 μg/mL) at 37 °C for 2 h. **B** The number of viable bacteria in biofilms after treatment with different concentrations of Ply113 (0–16 μg/mL) at 37 °C for 2 h. The data are presented as the mean ± SD from three independent tests with three biological replicates for each sample. Statistical significance was calculated via one-way ANOVA. ***p < 0.001. **C** CLSM imaging of SYTO9–PI-stained *S. suis* biofilms following treatment with Ply113 at a concentration of 16 μg/mL. **D** COMSTAT analysis of the corresponding biofilm CLSM images. **E** CLSM imaging of *S. suis* biofilms stained with Alexa Fluor 350-labelled Concanavalin A (exopolysaccharides, blue) in 24-h-established biofilms followed by 2 h treatment of Ply113. Scale bar, 20 μm **F** Quantification of fluorescence area of exopolysaccharides by ImageJ analysis. ***p < 0.001 significance was determined by comparison to the untreated control. Error bars represent the standard deviation of the data set (*n* = 4).

To investigate the antibiofilm activity of Ply113, the *S. suis* JL8F strain was chosen because of its ability to produce strong biofilms. A crystal violet staining assay was performed to examine the ability of Ply113 to eliminate established biofilms. As shown in Figure 3A, Ply113 effectively reduced the biomass of 24 h-old biofilms established by *S. suis* in a concentration-dependent manner. Treatment with 4 µg/mL Ply113 at 37 °C for 2 h eliminated 66.9% of the biofilm biomass, whereas treatment with 16 µg/mL Ply113 eliminated nearly all the biofilm biomass (93.2%). In addition, treatment with Ply113 for 2 h reduced the number of viable bacteria in *S. suis* biofilm, with a 0.98 log_10_ reduction at 4 µg/mL and a 2.19 log_10_ reduction at 16 μg/mL (Figure 3B).

The effects of Ply113 on the biofilms of the *S. suis* JL8F strain were evaluated by the CLSM observation. CLSM images showed that *S. suis* JL8F without Ply113 treatment formed a mature biofilm with an intact multilayered three-dimensional structure (Figure 3C). After 2 h of treatment, Ply113 efficiently eliminated 24-h-old biofilms, as evidenced by the structural destruction of the majority of the biofilms (Figure 3C). The bio-volume and thickness of *S. suis* biofilm were assessed using COMSTAT software. The analysis showed that the Ply113-treated biofilm exerted a decrease in biomass and mean thickness when compared to the control biofilm (Figure 3D). Furthermore, exopolysaccharides, as key constituents of the biofilm extracellular matrix, are essential for maintaining mature biofilm structure. To determine the effect of Ply113 on *S. suis* biofilm extracellular matrix, *S. suis* biofilms after Ply113 treatment were stained by Alexa Fluor 350-labeled Concanavalin A to visualize the biofilm exopolysaccharides. As shown in Figures 3E and F, there was a substantial reduction in the contents of exopolysaccharides in the biofilms treated with Ply113 compared to the control biofilm. These results suggested that Ply113 could eliminate *S. suis* biofilms through killing biofilm-derived cells and disrupting the extracellular matrix.

### Ply113 shows no toxicity to C57BL/6 mice

**Figure 4:**
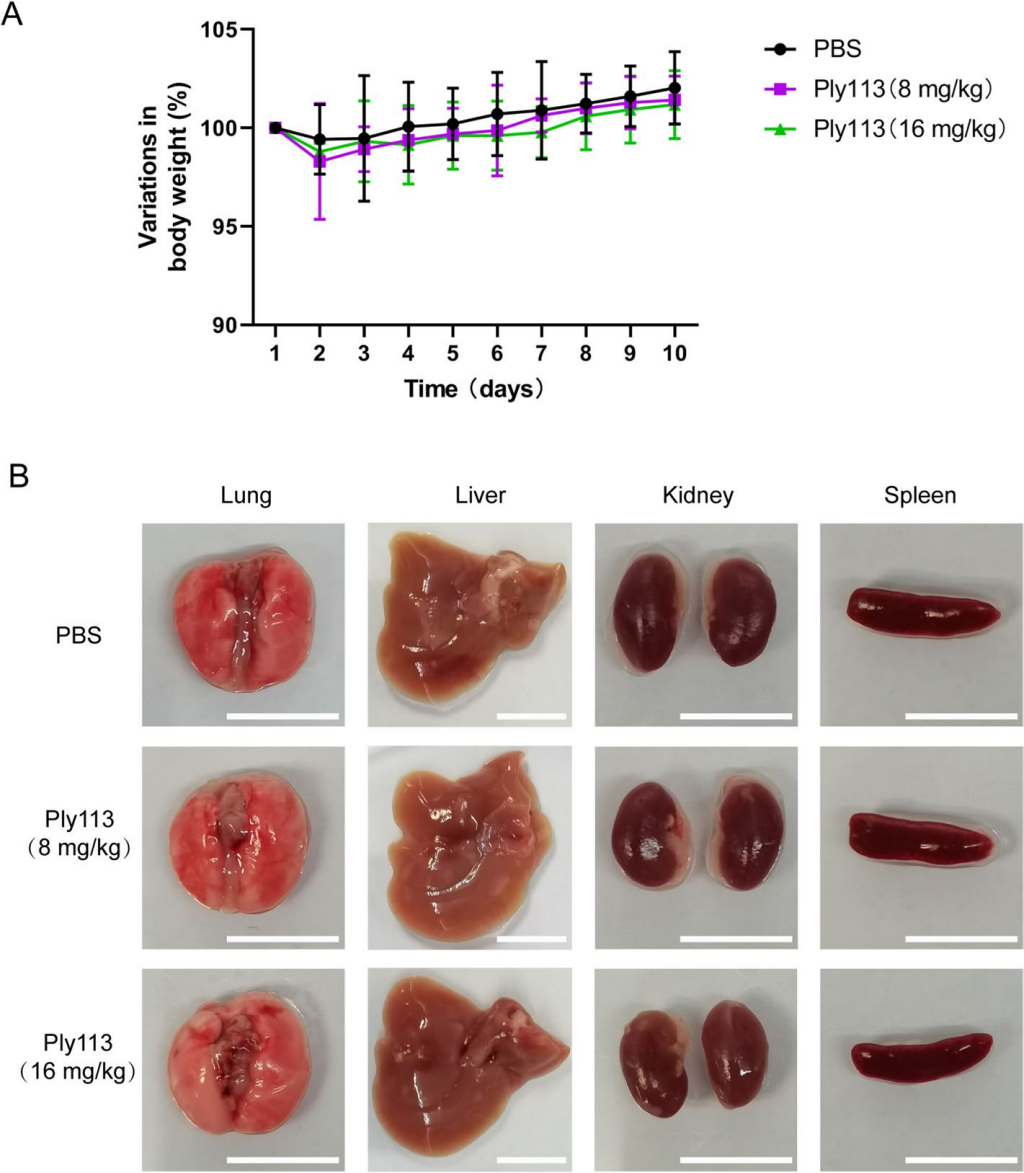
**Toxicity of Ply113 to mice**. **A** Variation in the body weight of the mice after P113 administration. The body weights of the mice in each group were recorded over 10 days after intraperitoneal injection of Ply 113 (8 and 16 mg/kg). The data are presented as the mean ± SD. **B** Organs of mice after treatment with Ply 113 (8 and 16 mg/kg) or PBS. Scale bars, 10 mm. No gross lesion changes were observed in the lungs, livers, kidneys, or spleens of the mice from each group.

To test the toxicity of Ply113 to mice, 6-week-old healthy C57BL/6 mice were intraperitoneally administered the therapeutic dose (8 mg/kg) or twice the therapeutic dose (16 mg/kg) of Ply113, and their overall health was evaluated for ten days. Neither the 8 mg/kg nor the 16 mg/kg doses caused death during the monitoring period. All the mice in each group maintained normal health and diet, as evidenced by the progressive increase in body weight over the observation period (Figure 4A). The organs of the mice in each group were collected 10 days after injection to determine whether they had incurred any toxic damage. As shown in Figure 4B, no gross lesions were observed in the lungs, livers, kidneys, or spleens of the mice injected with either the 8 mg/kg or the 16 mg/kg dose. These results suggested that Ply113 had no adverse effects on the health status of the mice.

### Ply113 rescues mice against lethal *S. suis* infection

**Figure 5:**
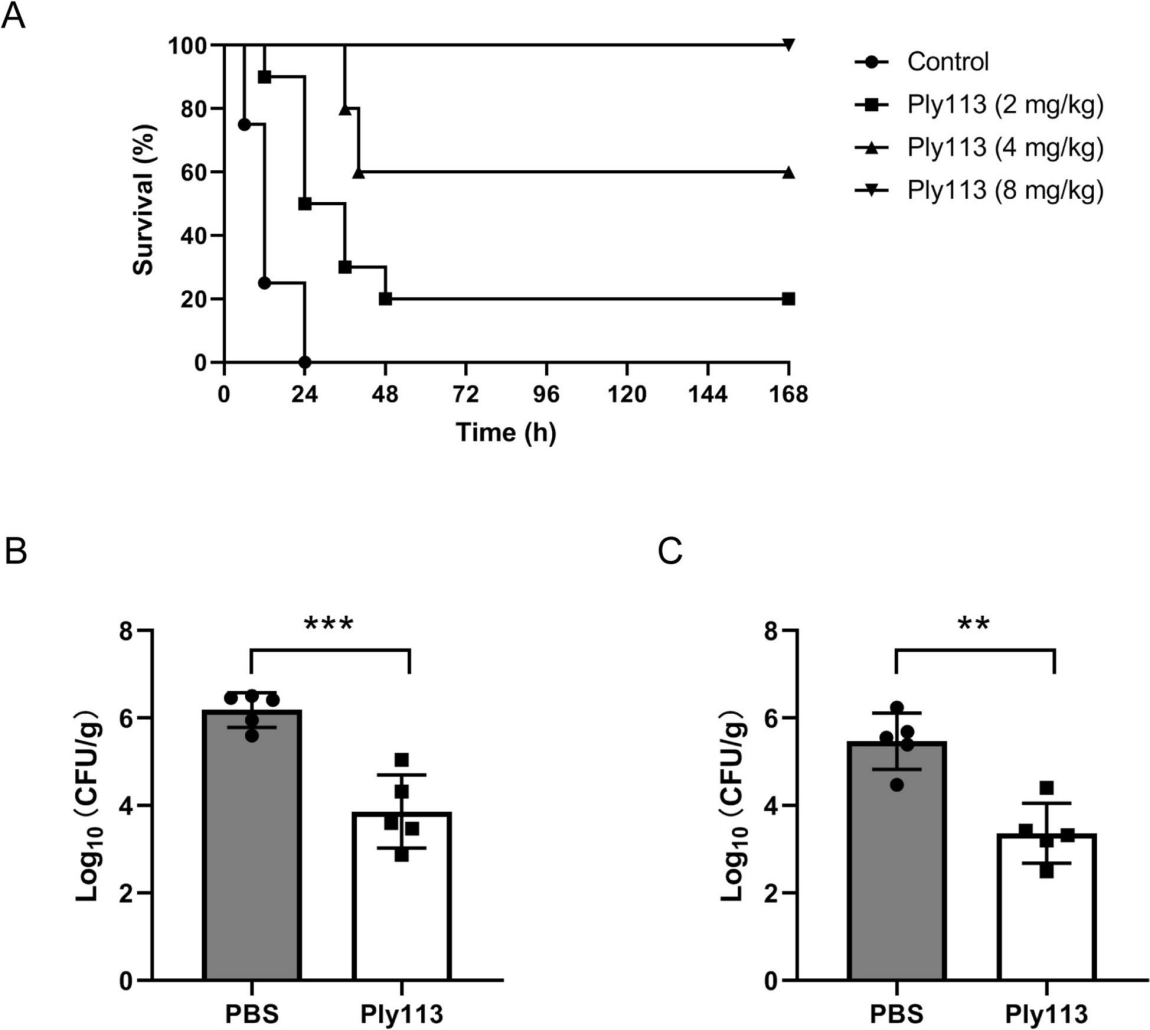
**Therapeutic efficacy of Ply113 in a mouse bacteremia model.** **A** Survival of mice infected with *S. suis* 05ZYH33 and treated with various doses of Ply113 (2, 4, and 8 mg/kg). Bacterial loads in (**B**) livers and (**C**) spleens from mice infected with *S. suis* and treated with 8 mg/kg Ply113. The data are presented as the means ± SD from five mice. Statistical significance was calculated via unpaired* t* tests. **p < 0.01; ***p < 0.001.

The in vivo therapeutic potential of Ply113 was evaluated using a murine bacteremia model. Intraperitoneal injection of 8 × 10^8^ CFU/mouse of *S. suis* 05ZYH33 was sufficient to cause 100% mortality within 24 h post-infection. Mice infected with *S. suis* for 2 h were treated with various doses of Ply113 (2, 4, and 8 mg/kg). All the mice in the control group treated with PBS died within 24 h post-infection. However, Ply113 displayed a dose-dependent therapeutic effect (Figure 5A). Treatment with a single dose of Ply113 (2 mg/kg) protected 20% of the mice, whereas injection of 4 mg/kg Ply113 rescued 60% of the mice. Ply113 at a dose of 8 mg/kg was capable of completely rescuing the mice from death, with a 100% survival rate (Figure 5A).

### Ply113 reduces bacterial burden and attenuates organ injury in *S. suis*-infected mice

The in vivo antibacterial efficacy of Ply113 was further examined by comparing the bacterial burdens in the lungs and spleens of the mice in the PBS- and Ply113-treated groups. C57BL/6 mice were infected with 8 × 10^7^ CFU of *S. suis* 05ZYH33 and treated with 8 mg/kg Ply113 or PBS as controls. After 24 h of treatment, the liver and spleen from each mouse were excised and homogenized for bacterial burden quantification. As shown in Figures 5B and C, bacterial counts in the liver and spleen were significantly different between mice treated with a single dose of Ply113 and those injected with PBS. Compared with the PBS control, Ply113 treatment reduced bacterial counts in the livers of *S. suis*-infected mice by 2.32 log_10_ (Figure 5B). In addition, the bacterial loads in the spleens of Ply113-treated mice decreased by 2.10 log₁₀ (Figure 5C).

**Figure 6:**
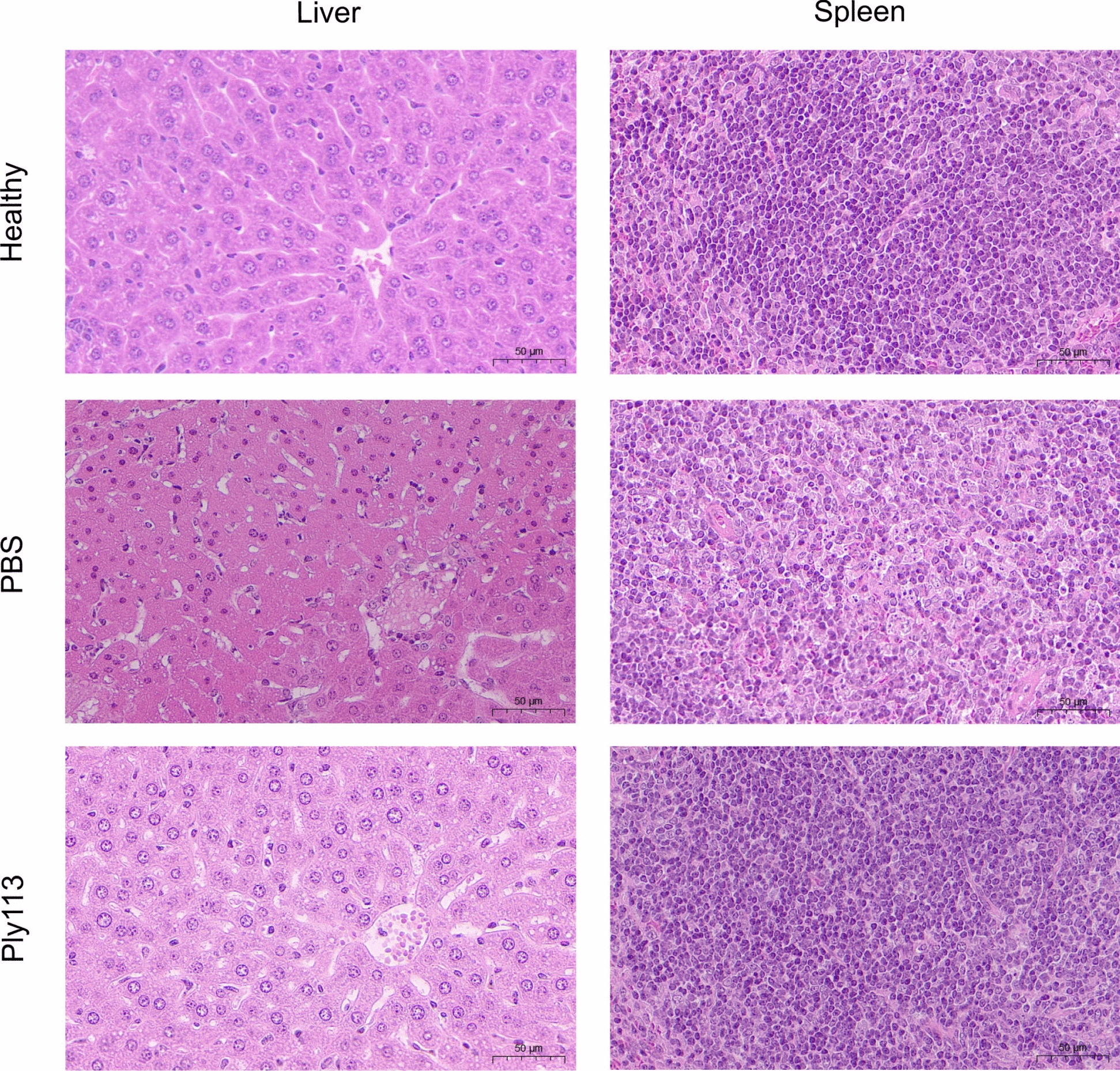
**Histopathological evaluation of organ injury in *S. suis*-infected mice treated with Ply113**. Representative H&E staining images of livers and spleens from healthy mice and infected mice subjected to PBS or Ply113 treatment. Scale bar, 50 μm.

Histopathological analysis was conducted to evaluate the severity of tissue lesions in mice treated with Ply113 and PBS. As shown in Figure 6, healthy mice had normal tissue morphology, with no noticeable lesions. However, H&E-stained sections revealed severe pathological damage to the tissues of *S. suis*-infected mice treated with PBS. The predominant pathological damage comprises multifocal patchy necrosis in the liver with extensive zones of hepatocyte degeneration and necrosis, as well as lymphoid depletion in the white pulp of the spleen, which manifests as diminished cellularity and lymphocyte necrosis (Figure 6). Compared with PBS treatment, Ply113 treatment noticeably alleviated the damage to the liver and spleen, with no obvious pathological injury detected (Figure 6).

## Discussion

*S. suis* infection is a primary contributor to mortality and substantial economic losses in the pig industry worldwide. *S. suis* has a global distribution. Its prevalence exceeds 40% of pig farms in China, Canada, and Vietnam, and rises above 50% in Korea and Thailand [31, 32]. Of particular concern is the increasing global trajectory of antibiotic resistance in *S. suis*. Currently, *S. suis* has limited resistance rates to β-lactams, aminoglycosides, fluoroquinolones and sulfonamides, but high resistant rates (> 80%) to tetracyclines, macrolides, and lincosamides worldwide [12, 33]. Alarmingly, multidrug-resistant (MDR) strains now account for more than 90% of clinical isolates [12], highlighting the critical necessity for developing novel therapeutic strategies.

Phage-derived proteins, particularly endolysins, are emerging as groundbreaking therapeutic weapons against antimicrobial resistance. Our previous study demonstrated that Ply113, an endolysin derived from an *E. faecium* phage, exhibits potent bactericidal activity against notorious antibiotic-resistant pathogens, including *E. faecium*, *E. faecalis*, and *S. aureus* [22]. The remarkable cross-species lytic capability of Ply113 prompted our investigation into its efficacy against *S. suis* in order to address its severe impact on swine health. In the present study, we found that Ply113 also exhibited potent bactericidal activity against *S. suis* strains. To date, several endolysins from enterococcal phages have been characterized and exhibited different lytic spectra. LysEFm5A, a lysin derived from an *E. faecium* bacteriophage, showed potent antibacterial activity against *E. faecium* but no observable activity against other tested bacterial species [34]. Similarly, the LysEF-P10 lysin exhibited efficient bactericidal activity against *E. faecalis* strains while showing no efficacy against *E. faecium* [35]. The ORF9 endolysin, derived from an *E. faecalis* phage, exhibited lytic activity against both *E. faecalis* and *E. faecium* but showed no activity against *S. aureus* or *E. coli* strains [36]. In this study, the Ply113 endolysin exhibited potent bacteriolytic activity against three taxonomically distinct Gram-positive genera (*E. faecium*, *E. faecali*s, *S. aureus*, and *S. suis*), enhancing its therapeutic potential for controlling polymicrobial infections.

Importantly, biofilms play crucial roles in *S. suis* infections, contributing significantly to bacterial persistence and immune evasion. In the biofilm state, *S. suis* not only evades phagocytosis by host immune cells but also suppresses the formation of neutrophil extracellular traps through its biofilm matrix [37, 38]. Furthermore, compared with planktonic cells, biofilm-derived cells of *S. suis* was found to have a higher survival rate in host blood compared to planktonic cells [37]. Once established in vivo, *S. suis* biofilms often lead to chronic infections that are notoriously difficult to eradicate [39]. Notably, the majority of clinical *S. suis* isolates possess strong biofilm-forming capabilities [40], highlighting the urgent need for biofilm-targeted therapeutic strategies. Although several endolysins that are active against *S. suis* have been characterized, experimental data regarding their efficacy against *S. suis* biofilms remain limited [41–45]. A previous study showed that LySMP endolysin could effectively eliminate 80% of the established biofilms of *S. suis* [46]. Similarly, AVPL, an endolysin derived from *Aerococcus viridans* phage, has been found to be effective in both inhibiting and disrupting *S. suis* biofilms [47]. In our study, Ply113 exhibited potent antibiofilm activity, even at concentrations as low as 4 µg/mL. Notably, Ply113 at 16 µg/mL achieved nearly complete biofilm eradication. These findings are consistent with the limited existing reports on endolysin-mediated biofilm control in *S. suis*, collectively supporting the potential of endolysins as an effective strategy against biofilm-associated infection caused by *S. suis*.

Undoubtedly, safety represents a fundamental prerequisite for the clinical application of endolysins. Some preclinical investigations have consistently demonstrated that endolysins exhibit excellent safety properties, with no adverse effects in animal models [48–50]. This preclinical evidence has been further corroborated by human clinical trials. Specifically, the human phase 1 study of the SAL200 endolysin validated its safety, with participants experiencing only minor and temporary symptoms such as fatigue and myalgia, and no significant side events were noted [51]. However, the safety profiles of various endolysin classes may differ, and some may pose risks to the host [52]. To this end, we conducted safety assessments of Ply113 through high-dose administration in murine models. After receiving Ply113, the mice showed no observable signs of toxicity, maintained normal growth in body weight, and exhibited no histopathological alterations in major organs. These findings confirmed the favorable safety profile of Ply113, supporting its potential for therapeutic development. However, comprehensive safety assessments across multiple models are still needed. Another noteworthy concern in endolysin applications is their potential immunogenicity. Upon administration, endolysins may trigger immune responses, including the production of neutralizing antibodies and pro-inflammatory cytokines, which can significantly reduce their serum half-life [48, 53]. In clinical trials of the SAL200 endolysin, approximately 37% of subjects developed detectable antibodies following intravenous administration. Nevertheless, antimicrobial activity in blood was still observed at therapeutic doses not less than 1 mg/kg [51]. To address the issue of immunogenicity, researchers have proposed the strategy of engineering endolysins to improve their compatibility with human immune cells, thereby reducing host clearance of these heterologous proteins [53]. Our future research may involve modifying Ply113 using alternative chimeric protein designs to minimize immune responses in vivo.

The therapeutic potential of Ply113 was evaluated in a mouse model of *S. suis*-induced bacteremia. The therapeutic efficacy of Ply113 was dose-dependent, which is consistent with in vivo observations made with other endolysins, such as Cpl-1 and PlySK249 [54, 55]. In addition, Ply113 treatment significantly reduced bacterial loads in the liver and spleen of *S. suis*-infected mice, resulting in a reduction of more than 2 log_10_. Notably, a clinical trial using phage therapy for chronic otitis caused by *Pseudomonas aeruginosa* demonstrated that reducing the bacterial burden by 80% was sufficient for effective treatment [56]. Given that Ply113 achieved > 95% bacterial clearance in vivo within 24 h, it may be an ideal candidate for combating *S. suis* infections.

In summary, the present results demonstrate that the Ply113 endolysin exhibits potent and broad-spectrum bactericidal activity against *S. suis*, efficiently lysing the most prevalent serotypes of *S. suis* (2, 3, 4, 7, and 9). This endolysin can significantly disrupt established biofilms of *S. suis*. Furthermore, a single administration of Ply113 significantly reduces the bacterial burden in systemically infected mice, alleviates organ pathological injury, and provides complete protection against lethal *S. suis* infections. These findings position Ply113 as a promising enzybiotic candidate for combating systemic *S. suis* infections. Future studies should focus on the optimization of Ply113 production and comprehensive preclinical evaluation in porcine models to advance therapeutic development.

## Data Availability

All data supporting the findings of this study are included in the article. Additional data are available from the corresponding authors upon request.
